# Microsatellite Genotyping and Genetic Diversity of a Greek Pear (*Pyrus communis* L.) Germplasm Collection

**DOI:** 10.3390/plants14121816

**Published:** 2025-06-13

**Authors:** Eleftheria Deligiannidou, Anastasia Boutsika, Ioannis Plesias, Aliki Xanthopoulou, Theodoros Moysiadis, Ifigeneia Mellidou, Ioannis Manthos, Thomas Sotiropoulos, Ioannis Ganopoulos

**Affiliations:** 1 Institute of Plant Breeding and Genetic Resources, ELGO DIMITRA, Thermi-Thermi Road, 570 01 Thermi, Thessaloniki, Greece; rikadel22@gmail.com (E.D.); bouanastasia@outlook.com (A.B.); giannesplesias@gmail.com (I.P.); aliki.xanthopoulou@gmail.com (A.X.); moysiadis.t@unic.ac.cy (T.M.); imellidou@elgo.gr (I.M.); jmanthos@elgo.gr (I.M.); 2 Department of Computer Science, School of Sciences and Engineering, University of Nicosia, Nicosia 2417, Cyprus; 3 Institute of Plant Breeding and Genetic Resources, Department of Deciduous Fruit Trees, ELGO-DIMITRA, R.R. Station 38, 59200 Naoussa, Greece; thsotiropoulos@elgo.gr

**Keywords:** microsatellites, genotyping, pear, Gene Bank collection, cluster analysis, genetic diversity

## Abstract

Pear (*Pyrus communis* L.) is a widely cultivated fruit tree species, valued for its significant economic impact and cultural relevance. The rise in commercial cultivars, characterized by genetic uniformity and high yield, is increasingly displacing traditional landraces. However, traditional varieties are highly adapted to local environmental conditions, having resulted from centuries of selection. In this study, 51 pear (*Pyrus communis* L.) accessions conserved in the Greek national germplasm collection were genotyped using eight SSR markers recommended by the European Cooperative Programme for Plant Genetic Resources (ECPGR). A total of 44 alleles were detected, including several private alleles, indicative of localized adaptation or potential genetic isolation. Analyses of population structure and genetic diversity, using Principal Coordinate Analysis (PCoA), UPGMA clustering, and Bayesian inference via STRUCTURE, uncovered distinct genetic groupings within the collection. The results revealed moderate genetic variability among the 51 accessions and identified some accessions with significant genetic divergence. These findings underscore the importance of conserving Greek pear germplasm, as it represents an ideal source of desirable traits, such as stress tolerance and fruit quality, which can be utilized in breeding programs.

## 1. Introduction

The pear (*Pyrus communis* L.), a member of the Rosaceae family, is a popular fruit known for its delicate taste and pleasant aroma. It is one of the oldest cultivated crops in the world, dating back almost 3000 years. The species is believed to have originated in the hilly regions of southwest China [1,2]. Cultivated species of the genus are primarily diploid and triploid, with a basic chromosome number of x = 17. Pears are highly valued as important temperate fruit trees [3,4,5]. However, human activities, agricultural practices, and urban development threaten wild *Pyrus* populations [6]. As a result, cultivated local wild varieties are at risk of extinction [7] or are being replaced by modern ones. This phenomenon reduces genetic variability and contributes to genetic erosion [8] as modern, high-yielding commercial varieties increasingly dominate the market [3,9].

Εx situ germplasm collections maintained in Gene Banks can preserve natural genetic materials with valuable characteristics [10,11], on which plant breeding programs heavily rely [12]. The Agricultural Research Service (USDA-ARS) of the US Department of Agriculture maintains the world’s largest pear gene bank in Oregon, with over 2500 distinct clones and seedlings [11,13]. In Greece, the only recognized repository for *Pyrus* germplasm is the Department of Deciduous Fruit Trees of the Institute of Plant Breeding & Genetic Resources (ELGO DIMITRA). Its pear collection includes both commercial cultivars and older accessions with valuable agronomic traits, including disease resistance and drought tolerance.

Gene banks are essential for safeguarding the rich diversity of pear varieties. Beyond their historical and cultural significance, older varieties, known as landraces, may harbor unique genetic resources that remain largely unexplored by breeders. These landraces can offer a valuable gene pool and hold the potential to improve future varieties by providing genes related to environmental stresses, longer shelf life, earlier ripening, and unique characteristics such as red-fleshed fruit, even though they might not always display superior commercial traits [14]. Due to the region’s unique environmental conditions and centuries of traditional cultivation, Greek pear germplasm stands out as a distinct genetic pool. Thus, our study emphasizes the uniqueness of Greek germplasm as a genetic resource and highlights the impact of local, environmental, and historical factors on its development. Its distinction from the broader USDA pear collection, along with its genetic characterization, is crucial to preventing genetic erosion and to preserving pear biodiversity at the national level [15,16].

Numerous studies have characterized national germplasm collections of pears in various countries, including Spain [16,17,18], Italy [19], Sweden [20], Germany, and Poland [21]. These studies reveal that the genetic diversity of pears can be effectively estimated using comprehensive Simple Sequence Repeats (SSR) analysis [11,20,22,23,24,25,26,27,28,29,30], as SSR markers are reproducible, multiallelic, codominant, abundant, and provide good genome coverage [31].

In this study, we assessed the genetic diversity of Greek pear cultivars from the Greek National Collection using SSR markers, in accordance with the guidelines of the European Cooperative Program for Plant Genetic Resources (ECPGR). The main objective was to gain insight into the genetic identity of 51 pear accessions from this collection and to apply this knowledge to breeding programs and conservation initiatives.

## 2. Materials and Methods

### 2.1. Plant Material and DNA Extraction

A total of 51 accessions of *P. communis* were analyzed in this study (Table 1). Among them, there were 6 local varieties, 16 breeding lines, and 29 international cultivars. Samples were collected from their historical regions of origin and have been maintained in the Greek National Pear Collection for over 10 years. The collection is located at the Institute of Plant Breeding and Genetic Resources (IPGRB)-Department of Deciduous Fruit Trees, ELGO DIMITRA, in Naoussa.

Young leaves from each variety were collected, snap-frozen, and stored at −20 °C until DNA extraction. Genomic DNA was extracted using the Higher Purity™ Plant DNA Purification Kit (Canvax Biotech, Valladolid, Spain), following the manufacturer’s instructions.

### 2.2. Microsatellite Genotyping

A set of 8 microsatellite markers were selected for genotyping. Two multiplex PCR assays were designed, each containing a panel of SSR markers. The fluorophores used for labeling the SSR markers were FAM, ROX, TAMRA, and HEX, which facilitated the simultaneous detection of multiple loci within a single reaction. The details of the SSR markers [20,32,33,34,35,36], including their names, dye labels, amplicon size ranges (min-max), and sequences of forward (F) and reverse (R) primers, are listed in Table 2 for Multiplex 1 and Multiplex 2. Multiplex Set 1 includes the primers EMPc117 (Tm: 61.8 °C, Dye: FAM, Allelic Range: 85–135 bp), CH01d08 (Dye: FAM, Allelic Range: 277–301 bp), EMPc1 (Dye: TAMRA, Allelic Range: 135–155 bp), CH01f07a (Dye: TAMRA, Allelic Range: 175–211 bp), and CH05c06 (Dye: ROX, Allelic Range: 83–111 bp). Multiplex Set 2 includes CH04e03 (Dye: FAM, Allelic Range: 179–221 bp), CH03g07 (Tm: 60.8 °C, Dye: HEX, Allelic Range: 195–265 bp), and GD147 (Dye: HEX, Allelic Range: 121–147 bp). These combinations were selected based on compatibility in melting temperatures, non-overlapping allelic size ranges, and distinct fluorescent dye labeling to ensure accurate and efficient fragment analysis.

Ninety-eight PCR reactions were performed in total. Each reaction had a total volume of 10 μL, consisting of 5 μL of KAPA2G Fast Multiplex PCR Mix (2×), 1 μL of template DNA (100 ng/μL), 0.2 μL of each forward (10 mM), and 0.2 μL of each reverse primer (10 mM) mix. Finally, nuclease-free water was added to reach the final volume of 10 μL. The thermal cycling conditions for PCR amplification were optimized for each multiplex and are summarized below. For Multiplex 1: Initial denaturation at 95 °C for 5 min, denaturation at 95 °C for 30 s, annealing at 61.8 °C for 30 s, and extension at 72 °C for 30 s, repeated for 35 cycles, with a final extension at 72 °C for 10 min. For Multiplex 2: Initial denaturation at 94 °C for 5 min, denaturation at 94 °C for 30 s, annealing at 60.8 °C for 45 s, and extension at 72 °C for 1 min, repeated for 30 cycles, with a final extension at 72 °C for 7 min.

The PCR products were separated and detected using capillary electrophoresis. Fragment analysis was conducted in an ABI 3730xl (Applied Biosystems, Foster City, CA, USA) with GeneScan 500 LIZ size standard, and results were recorded with GeneMapper v4. Cultivar reference samples were genotyped twice to avoid sample mix-up (two independent DNA extractions and PCR amplifications). PCR amplifications were repeated for a random sample of 5 individuals (~10% of the whole dataset). The data were used to calculate the error rate (~2%): (i) per reaction and (ii) per allele [37].

To test whether the eight microsatellite loci were informative enough to distinguish the pear accessions, statistical re-sampling showed that these microsatellite loci were sufficient to ensure identification. According to the discriminating power value for each locus, we tested combinations starting with the most discriminating and adding one locus at each step. The optimal combination (CH03g07 + CH04e03 + GD147 + CH01d08 + CH01f07a + CH05c06 + EMPc11 + EMPc117) successfully discriminated all analyzed accessions analyzed. Using this locus combination, we observed a low probability of identity (PI = 3.9 × 10^−5^; Table 2). Also, for the *Pyrus* genus, as few as six microsatellite markers can be sufficient to facilitate cross-comparisons between collections in order to detect duplicates and synonyms with minimal chance of misidentifying a genotype with a randomly selected one from a larger sample [38].

### 2.3. Data Analysis

Genetic diversity estimates, including number of alleles (Na), private alleles (Np), Shannon’s index (I), and expected heterozygosity (He), were calculated using GenAlEx 6.51b2 software [39]. Polymorphic Information Content (PIC) values for each microsatellite marker were calculated using PowerMarker v3.25 software [40] to assess the discriminatory power of different SSR primers. The probability of identity (PI) measures the probability that two randomly drawn diploid genotypes will be identical, assuming the observed allele frequencies and random assortment [41]. The total probability of identity, defined as the probability of two cultivars sharing the same genetic profile by chance, was also calculated from the individual PI values. PI was calculated by IDENTITY 1.0 (Centre for Applied Genetics, University of Agricultural Sciences, Vienna, Austria). The allelic data generated by fragment analysis were examined with GenAlEx [39], STRUCTURE program [42], and RStudio version 4.3.1 [43] to assess genetic diversity, population structure, and other significant characteristics. The R packages used for the genetic analysis were ape [44], phangorn [45], readr [46], ggplot2 [47], ggrepel [48], cluster [49], factoextra [50], and NbClust [51]. A Principal Coordinate Analysis (PCoA) was conducted to evaluate genetic relationships among the 51 pear accessions, which included both local and commercial cultivars, using both GenAlEx and R for complementary visualizations. Specifically, it was conducted on the SSR genotypic matrix, formatted specifically for compatibility with GenAlEx (Genetic Analysis in Excel) software [39] to determine the proportion of variation explained by each primary coordinate, thus allowing the multivariate statistical analysis of many variables [7,52]. Furthermore, a pairwise Euclidean distance matrix was calculated from the scaled genotype matrix, utilizing the classical multidimensional scaling function cmdscale() from the base R stats package [43]. Eigenvalues were obtained to determine the proportion of variance attributed to each principal coordinate axis. The PCoA scatterplot was generated utilizing ggplot2 [47], with sample points distinguished by population grouping and selectively annotated through the ggrepel package [48]. A scree plot was created to illustrate the variance accounted for by the initial 13 axes. All analyses were conducted utilizing R version 4.x.

Hierarchical clustering was conducted using the Unweighted Pair Group Method with Arithmetic Mean (UPGMA) to investigate the genetic structure of a single pear population. The analysis utilized a Euclidean distance matrix obtained from standardized SSR marker data. The preprocessing steps involved averaging the values of duplicated markers, substituting missing values (coded as -9) with the mean of the respective marker column, and eliminating loci exhibiting no variation. The dataset underwent standardization through z-score transformation to normalize markers and ensure uniform weighting. A Euclidean distance matrix was generated from the scaled data utilizing the dist() function in R. UPGMA clustering was performed utilizing the hclust() function, with the method specified as “average,” aligning with UPGMA, as described by Sneath and Sokal [53]. The hierarchical clustering result was transformed into a phylogenetic tree object utilizing the as.phylo() function from the ape package [54], facilitating enhanced formatting and visualization of the dendrogram.

All samples originated from a single population and were represented using a consistent color scheme for tip labels. The dendrogram was exported in high resolution via the png() graphics device, with enhanced font sizes and spacing to ensure clarity in label presentation. This method facilitated the identification of genetic subgroups and outlier genotypes within the examined population.

The ‘admixture’ and ‘independent allele frequencies’ models were used to run STRUCTURE 2.3.4 [42]. A burn-in of 200,000 iterations and 500,000 MCMC repetitions for each run were executed, with 20 replicates from K = 1 up to K = 8. The CLUMPAK main pipeline [55] was used to merge replicate runs, and the optimal K value was inferred using Evanno’s method [56], which was run in the pophelper 2.3.0 R package [57]. The software Structure threader [58] was used to parallelize computations. The terms “populations” and “subpopulations” used throughout the manuscript refer exclusively to the genetic clusters inferred from STRUCTURE analysis. No a priori grouping was applied; the structure was inferred de novo based on multilocus genotype data using the admixture model.

## 3. Results

The total number of distinct alleles (Na), alleles with a frequency ≥ 5% (Na Freq. ≥ 5%), effective number of alleles (Ne), Shannon’s Information Index (I), expected heterozygosity (He), and its unbiased version (uHe) were among the key parameters used to evaluate the genetic variation in *P. communis* genotypes across various loci (Table 3). Private alleles and the presence of locally common alleles (No. LComm Alleles) were also assessed to provide insights into genetic diversity and population structure (Table 4).

The entire collection of IPGRB accessions was used, and across all loci, a total of 44 distinct alleles (Na) were detected, with 21 of these having a frequency of ≥5%. The effective number of alleles (Ne), a measure of allelic diversity, was also 44, reflecting a moderate level of genetic diversity within the collection. Nevertheless, neither 25% nor 50% of the population showed evidence of regionally common alleles, suggesting limited gene flow or exchange between subpopulations and indicating potential genetic isolation within specific regions. Averaged across all loci, each genetic locus exhibited approximately 5.5 distinct alleles (Na), with 2.6 alleles occurring at frequencies ≥ 5% and a range of 2 (locus CH03g07) to 13 (locus EMPc117). Although the total number of alleles detected was 44, the effective number of alleles (Ne) averaged 2.34, suggesting that, despite the presence of a variety of alleles, only a smaller subset contributes effectively to the genetic variation within the collection, meaning that some alleles were rare and contributed little to diversity.

Shannon’s Information Index (I) averaged 0.953 across loci, indicating a moderate degree of genetic diversity, as a higher value would imply greater genetic variety. On average, the expected heterozygosity (He), which estimates the probability that two randomly selected alleles are different, was 0.505, while the unbiased expected heterozygosity (uHe) had a similar value of 0.510. These heterozygosity values suggest a moderate level of genetic variability within the collection. The total value of the probability of identity was a total of 3.9 × 10^−5^.

Notably, no regionally common alleles were detected at any significant frequency, further supporting the notion that the collection is genetically diverse but exhibits limited allele sharing among its subgroups. This is also reflected in the presence of several private alleles associated with specific accessions that clustered separately and belonged to certain subpopulations, indicating that some genotypes possess distinctive allelic combinations not widely distributed across the collection.

**Figure 1:**
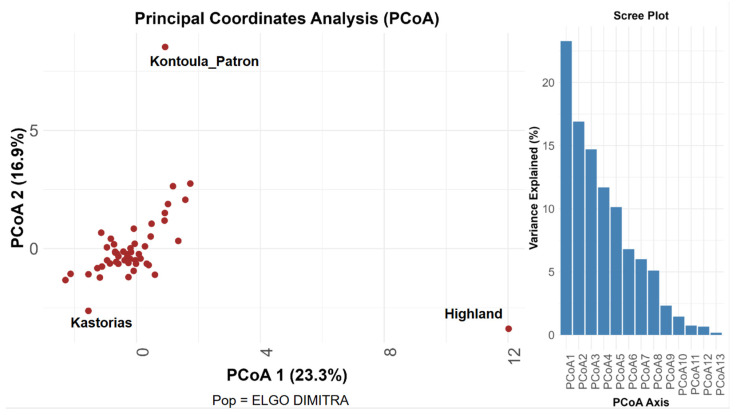
PCoA analysis. Principal Coordinate Analysis (PCoA) based on SSR marker data from 51 pear (*Pyrus communis* L.) accessions. The plot displays the first two coordinate axes, PCoA 1 and PCoA 2, which together explain 40.2% of the total genetic variation (23.3% and 16.9%, respectively). The corresponding scree plot illustrates the variance explained by each axis.

Principal Coordinate Analysis (PCoA), utilizing pairwise genetic distances among 51 pear accessions, revealed a distinct genetic structure. A total of 40.2% of the genetic variance was explained by the first two PCoA axes, with PCoA1 contributing 23.3% and PCoA2 contributing 16.9% (Figure 1). The clustering of the majority of individuals in the center of the PCoA plot indicates minimal genetic differentiation among the accessions. However, three genotypes, ‘Highland’, ‘Kontoula_Patron’, and ‘Kastorias’, located outside the central cluster, suggest increased genetic divergence. ‘Highland’ showed significant separation along the PCoA1 axis, whereas ‘Kontoula_Patron’ demonstrated a clear distinction along PCoA2. ‘Kastorias’ was positioned on the far left of the plot, deviating from both axes. This distinction highlights their potential singularity within the broader collection. This conclusion was supported by the scree plot, which indicates that the initial PCoA axes accounted for the majority of genetic variation, with eigenvalues declining significantly after the third axis. The most significant contributors to the first principal coordinate axis (PCoA1) among the examined SSR loci were found to be CH05c06 and EMPc117, whereas those to the second axis (PCoA2) were found to be CH01d08 and CH03g07. These loci exhibited the strongest associations with the spatial distribution of accessions in the PCoA plot, as derived from the coordinate loadings calculated in GenAlEx. The high influence of CH05c06 and EMPc117 aligns with their high allelic richness and variability, further supporting their discriminating power in revealing population structure.

**Figure 2:**
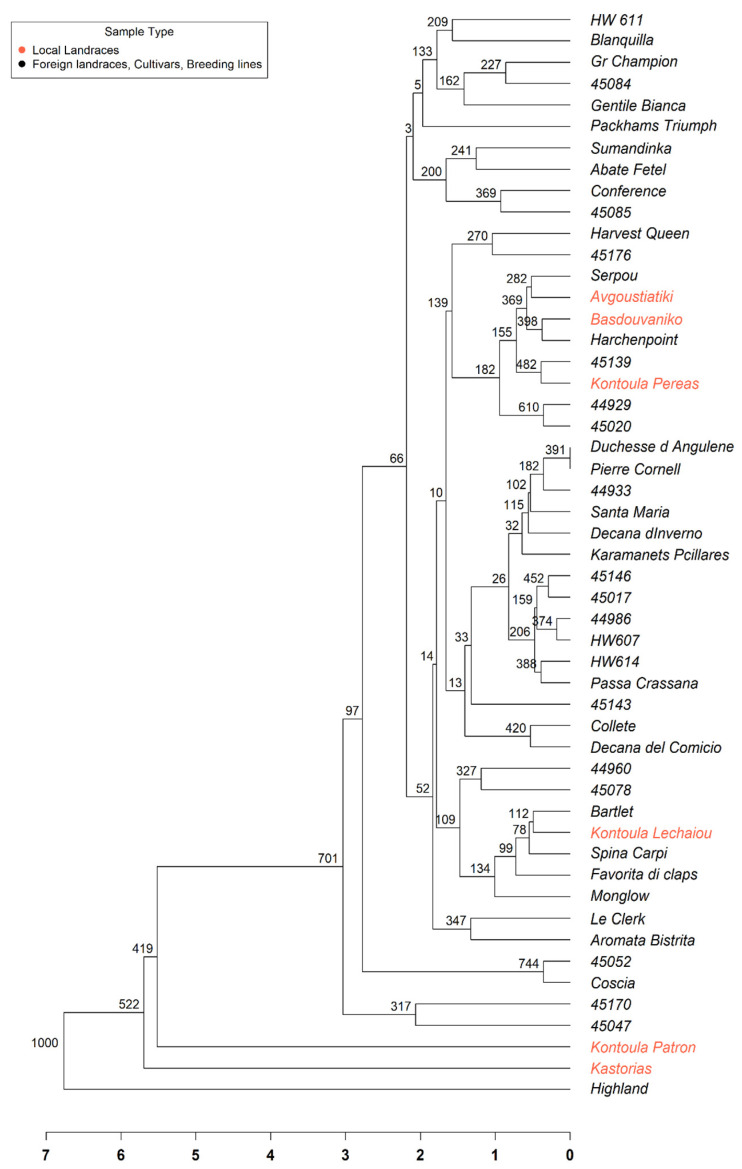
Unweighted Pair Group Method with Arithmetic Mean (UPGMA) dendrogram illustrating the genetic relationships among 51 pear (*Pyrus communis* L.) accessions based on SSR marker data. The branches with red color represent the Greek local landraces. The dendrogram was constructed using Euclidean genetic distances derived from standardized multilocus genotypic data. Bootstrap values and a scale bar are also shown.

The UPGMA dendrogram (Figure 2) demonstrated moderate genetic diversity among the accessions, even though they originated from a single population. Several genotypes, including ‘Kontoula Patron’, ‘Highland’, and ‘45047’, exhibited distinct and elongated branches, indicating greater genetic divergence from the remaining group. Furthermore, many accessions were grouped into closely related subgroups, suggesting a higher degree of genetic similarity. Despite the uniform color of all samples, the branching pattern revealed internal genetic differentiation. The tree structure provided valuable insights into the relationships among genotypes and can support future conservation and varietal selection efforts within this collection.

**Figure 3:**
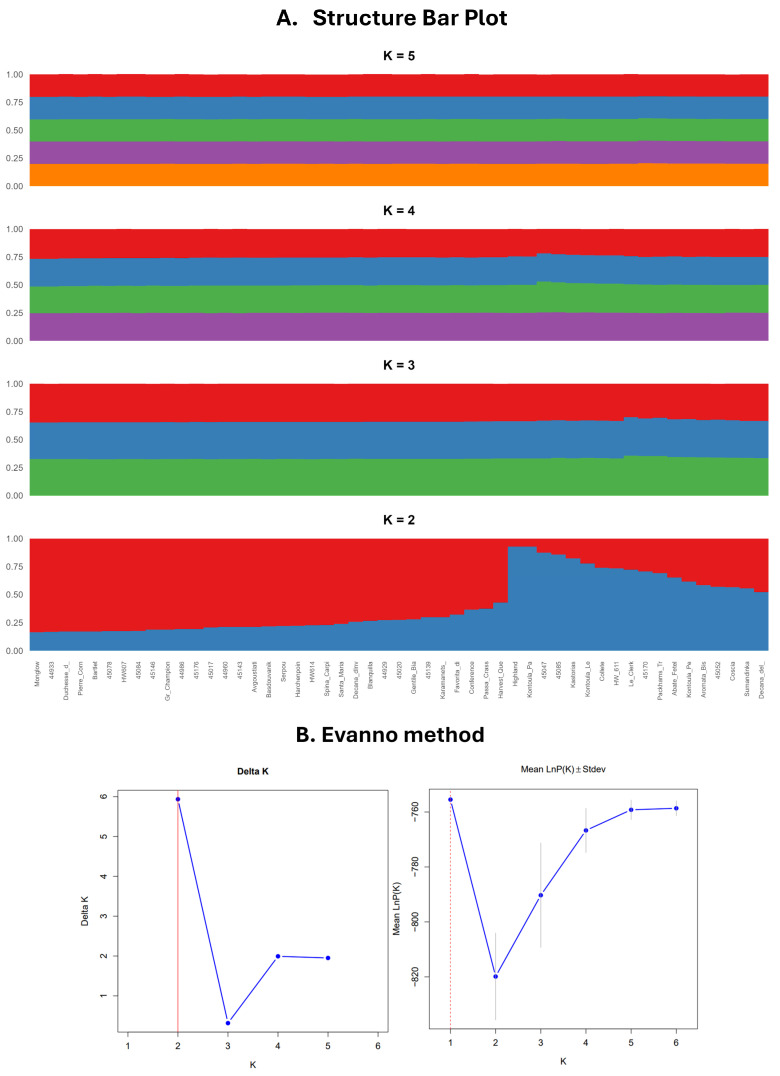
This figure presents the (**A**) STRUCTURE bar plot for K = 2, 3, 4, 5, revealing that the optimal number of genetic clusters is K = 2; (**B**) optimal cluster determination by the Evanno method. The ΔK method detected the highest rate of change at K = 2, and LnP(K) suggested increasing fit with higher K. The Evanno method corrected for overfitting and pointed to K = 2 as the most meaningful.

Analysis of population structure utilizing the Bayesian clustering method in STRUCTURE revealed that the optimal number of genetic clusters is K = 2 and identified two subpopulations, corroborated by the highest ΔK value (Figure 3). At this level, individuals were distinctly categorized into two primary groups. The initial group (Cluster 1, shown in blue) includes accessions such as Kontoula_Patron, Kastorias, and Highland, which were clearly classified with high membership coefficients. Cluster 2, represented in red, comprises the majority of the remaining samples, indicating a more genetically homogeneous group. Several accessions, including Aromata Bistrite, Le Clerk, and Spina Carpi, showed a notable presence in Cluster 1, despite previously being grouped with other accessions based on their geographic origin or cultivar classification. This pattern suggests potential admixture or a distinct genetic lineage, a phenomenon also reported in similar studies [11,23]. Subsequent STRUCTURE analyses at K = 3, 4, and 5 revealed further sub-structuring while maintaining consistency with the primary division observed at K = 2. Rather than indicating entirely new groupings, the higher K values revealed more diffuse patterns of differentiation, likely reflecting minor genetic variation resulting from cultivar development or local selection pressures.

The structure-based groupings received robust support from both Principal Coordinate Analysis (PCoA) and the UPGMA dendrogram, indicating the existence of two primary genetic groups within the dataset, with some individuals positioned at intermediate or divergent points. The PCoA plot (Figure 1) showed that Kontoula_Patron and Highland are distinct outliers along the PCoA1 axis, highlighting their genetic uniqueness. In line with the STRUCTURE results, the UPGMA dendrogram (Figure 2) distinctly clustered ‘Kontoula_Patron’, ‘Kastorias’, and ‘Highland’ apart from the main group, confirming their genetic divergence. Most genotypes from Cluster 2 belong to a robust clade, consistent with their shared ancestry.

## 4. Discussion

We characterized 51 pear accessions of the Greek Gene Bank collection using a set of eight SSR markers, which are broadly recommended by ECPGR and are suitable for the study of pear genetic resources [59]. The primary goal of this research was to gain insight into the genetic identity of the Greek collection of pears and utilize this information for breeding programs and conservation initiatives. The characterization of pear germplasm with SSR markers has already taken place for multiple pear collections worldwide (Table 5). In conservation genetic studies, where a large number of samples are studied, the use of SSR markers represents a suitable choice, as it is also the most economically beneficial option [60].

We have identified 44 distinct alleles across loci with an average of 5.5 alleles per locus. Regarding the number of alleles, by using a similar set of markers, other researchers came to different conclusions. Specifically, Queiroz et al. [23] characterized 54 Portuguese pear accessions with six SSR markers and detected 68 alleles with 11.3 alleles per locus; Sehic et al. [20] analyzed a European pear collection of 94 samples with 10 SSR markers and reported 104 alleles with 10.4 alleles per locus. Furthermore, Gasi et al. [22] studied 64 European pear accessions from Bosnia and Herzegovina using 13 microsatellite markers and found 159 alleles and 14.5 alleles per locus, while Kocsisné et al. [11] identified 216 alleles with 27 alleles per locus after the analysis of 88 cultivars from the Hungarian pear gene bank with eight SSR markers.

The effective number of alleles (Ne), with a value of 44, being equal to the total number of distinct alleles (Na) suggests that all detected alleles contribute to genetic diversity. However, with a mean of 2.43, it became clear that only a small portion of the total alleles contributed successfully to the genetic variation within the collection [61], which is similar to findings where the effective number of alleles was lower than the total number detected [18]. The mean of 5.5 distinct alleles per locus, ranging from 2 to 13, reflects the variability in genetic diversity across different loci, which is crucial for understanding the genetic structure and potential for breeding programs [11,26].

Considering the private alleles, the highest value (13) was found for the EMPc117 locus, while the lowest (2) was detected for the CH03g07 locus. Private alleles that are specific to a single wild pear collection can be used to estimate migration rates and quantify the genetic distinctiveness of the marker for the collections that are under investigation [62]. The presence of private alleles associated with specific accessions demonstrates that certain genotypes possess unique allelic combinations [27]. Furthermore, the average expected heterozygosity (He) was 0.505, which was relatively low compared to the mean values reported by Queiroz et al. [23,24] and Kocsisné et al. [11].

For specific loci, the effective number of alleles (Ne) and Shannon’s Information Index (I) varied from 1.104 and 0.229 (locus CH01d08) to 3.480 and 1.609 (locus CH05c06), respectively. In general, the CH01d08 locus displayed the lowest values among the loci examined and the CH05c06 locus suggested a high degree of genetic variety. This agrees with Queiroz et al. [23,24], who found similar values for this specific locus for the total (Na = 11.3, Na = 11) and effective (Ne = 3.3, Ne = 3.034) alleles, respectively. On the contrary, Kocsisné et al. [11] had nearly double these values for the CH05c06 locus. Generally, to improve the molecular identification of pear cultivars, it is necessary to have common guidelines among studies, because differing methodologies make it difficult to compare the results and create common databases [3]. Finally, there is also a need to include reference samples [11].

The PCoA effectively demonstrated a distinct genetic structure. The distinct positioning of the genotypes ‘Highland’, ‘Kontoula_Patron’, and ‘Kastorias’ outside the central cluster highlights their genetic divergence, further supporting the identification of separate evolutionary processes among different pear species, indicating that certain cultivars may have unique genetic backgrounds [30].

By performing the UPGMA phylogenetic analysis, we found moderate genetic diversity among the genotypes, which shows that genetic diversity can be significant even within a single population and suggests that environmental and geographic factors contribute to genetic differentiation [30]. Our pear germplasm showed a moderate level of genetic diversity overall, enough variation to support breeding and conservation efforts, while also containing some significantly divergent accessions, ‘Kontoula Patron’, ‘Highland’, and ‘45047’, that might be prioritized as reservoirs of unique alleles. This large genetic distance could also be due to hybridization with other Pyrus species, as reported by Bergonzoni et al. [14] for the cultivars ‘Cocomerina Selvatica La Casa’ (CS) and ‘Incrocio S. Alessio’ (IA). The determination of the genetic identity of these local cultivars is highly important for their conservation, as they adapt to distinct climatic conditions [8]. Understanding genetic relationships is crucial for maintaining diversity and selecting appropriate cultivars for breeding programs [30].

STRUCTURE analysis identified the optimal number of genetic clusters as K = 2, and this finding aligns with other studies, where similar Bayesian clustering methods revealed distinct genetic groupings within pear germplasm collections, supporting the utility of Bayesian methods in understanding population structure [63]. The clear categorization of accessions into two primary clusters reflects the genetic uniqueness of cultivars such as Kontoula_Patron and Highland, which may represent distinct genetic lineages [30]. The presence of significant admixture, where accessions such as Aromata Bistrite and Le Clerk were historically grouped with others based on geographic origin, suggests complex genetic relationships.

The results of the present study assessed the structure of the Greek pear germplasm collection and revealed valuable genetic diversity. The genetic-based clustering of the Greek pear germplasm collection uncovered high variability in pear cultivation in Greece, as well as the existence of plant material exchange between different regions. The presence of genetic variation is of utmost importance for a deeper understanding of the origin and evolution of traditional cultivars. Specifically, accessions carrying private alleles or showing unique genetic profiles may contain traits of agronomic importance, such as adaptation to local climatic conditions or resistance to biotic and abiotic stress factors, and the presence of private or rare alleles can be exploited in breeding programs. In addition, the use of microsatellite markers enhances the analysis of the genetic structure of the Greek pear germplasm collection. This acquisition of genetic data can contribute to the identification of duplicate entries, the verification of accession identity, and the development of a representative core collection. Given the impending climate change, which necessitates the utilization of biodiversity, all the above applications are of paramount importance for the conservation of Greek pear genetic resources. The present study serves as a springboard for the detailed phenotypic characterization and the implementation of traditional pear varieties in sustainable breeding programs.

**Table 5:** Data from other publications regarding the pear accessions and species, number of accessions, number of SSR markers, total number of alleles, Na (Mean number of alleles), Ne (Effective number of alleles), and He (Expected heterozygosity).

Publication	Pear Accessions	Pear Species	Number of Accessions	Number of SSR Markers	Total Number of Alleles	Na	Ne	He
[11]	Portuguese pear landraces	*P. communis*	88	8	216	27	-	0.88
[18]	Local Pear Cultivars (Aragon, Northeastern Spain)	*P. communis*, *P. spinosa*	108	9	162	18.11	8.45	0.83
[20]	Pear cultivars in Central Europe	*P. communis*	94	10	84	10.5	-	0.78
[22]	European pear (Bosnia and Herzegovina)	*P. communis*	64	13	159	14.5	-	-
[23]	Chinese National Pear Germplasm Repository (Wuhan)	*P. communis*	54	6	68	11.3	5.8	0.806
[24]	Pear collections	*P. communis*	130	11	129	11.7	5.8	0.79
[25]	Sardinian pears	*P.* spp.	19	21	-	-	-	0.3
[26]	“Zangli” pear landraces (Tibet)	*P.* spp.	67	28	202	7.21	4.07	0.72
[27]	Pear germplasm collection (Tunisia)	*P. pyrifolia*, *P. pashia*	478	17	121	7.12	6.36	0.78
[28]	Chinese National Germplasm Repository of Pear (Xingcheng, China)	*P.* spp.	131	17	377	22.17	7.77	0.86
[29]	Pear cultivars (Minas Gerais State, Brazil)	*P.* spp.	61	12	95	9.5	3.3	0.62
[30]	Collection of European pear cultivars	*P. communis*	252	14	251	17.93	6.83	0.82
[63]	Portuguese pear germplasm	*P.* spp.	385	134	690	5.45	-	0.74

## 5. Conclusions

Taken together, this study utilized eight SSR markers to identify an overall moderate genetic differentiation between the 51 *P. communis* accessions maintained in the national collection. Among the accessions, there were some significantly divergent ones, ‘Kontoula Patron’, ‘Highland’, and ‘45047’, which can be used as a source for unique alleles. The importance of this study for future breeding and conservation strategies is highlighted by the observed variations in expected heterozygosity and the presence of private alleles, which were detected in genetically distinct genotypes (e.g., ‘Highland’, ‘Kontoula Patron’) and whose presence, albeit in a conserved collection, likely reflects their origin from historically isolated environments. To gain more insight regarding genetic diversity and the possibilities for breeding enhancement, additional markers and a broader range of pear accessions should be selected and utilized for future research.

## Figures and Tables

**Table 1:** Pear accessions of Gene bank collection used in this study.

No	Accession Name	Origin	Group	No	Accession Name	Origin	Group
1	Duchesse d’Angulene	France	Cultivar	27	Bartlet	England	Cultivar
2	Pierre Cornell	France	Cultivar	28	Monglow	Maryland	Cultivar
3	Highland	England	Cultivar	29	Gentile Bianca	France	Cultivar
4	44960	Greece	Breeding line	30	Aromata Bistrita	Romania	Cultivar
5	45078	Greece	Breeding line	31	HW614	Serbia	Cultivar
6	45170	Greece	Breeding line	32	Passa Crassana	France	Cultivar
7	Kontoula Pereas	Greece	Landrace	33	45017	Greece	Breeding line
8	44929	Greece	Breeding line	34	45143	Greece	Breeding line
9	Serpou	France	Landrace	35	44986	Greece	Breeding line
10	45139	Greece	Breeding line	36	45146	Greece	Breeding line
11	Beurré d’Hardenpont	France	Cultivar	37	44933	Greece	Breeding line
12	Basdouvaniko	Greece	Landrace	38	Coscia	Italy	Cultivar
13	45020	Greece	Breeding line	39	Decana d’ Inverno	Belgium	Cultivar
14	Avgoustiatiki	Greece	Landrace	40	Decana del Comicio	France	Cultivar
15	Harvest Queen	Canada	Cultivar	41	Santa Maria	Italy	Cultivar
16	45176	Greece	Breeding line	42	Karamanets Pcillares	Bulgary	Cultivar
17	Blanquilla	Spain	Cultivar	43	45052	Greece	Breeding line
18	45047	Greece	Breeding line	44	Conference	England	Cultivar
19	Packham’s Triumph	England	Cultivar	45	45085	Greece	Breeding line
20	Colette	USA	Cultivar	46	HW607	Serbia	Cultivar
21	HW 611	Serbia	Cultivar	47	45084	Greece	Breeding line
22	Sumandinka	Serbia	Cultivar	48	Abate Fetel	France	Cultivar
23	Kontoula Patron	Greece	Landrace	49	Grand Champion	USA	Cultivar
24	Kontoula Lechaiou	Greece	Landrace	50	Le Clerk	France	Cultivar
25	Favorita di claps	USA	Cultivar	51	Kastorias	Greece	Landrace
26	Spina Carpi	Italy	Cultivar				

**Table 2:** Multiplex (M1 and M2) PCR assay primer characteristics. Linkage groups (LG) are reported according to the GDR database (https://www.rosaceae.org/) (Accessed on 1 March 2024).

	Primer	Dye	Repeat Motif	LG	Min	Max	Forward	Reverse	Tm	Bibliography
Multiplex 1	EMPc117	FAM	(CT)17	7	85	135	GTTCTATCTACCAAGCCACGCT	CGTTTGTGTGTTTTACGTGTTG	61.8	[20,36]
	CH01d08	FAM	(GA)n	3/15	277	301	CTCCGCCGCTATAACACTTC	TACTCTGGAGGGTATGTCAAAG		[32,34]
	EMPc11	TAMRA	(AC)13	11	135	155	GCGATTAAAGATCAATAAACCCATA	AAGCAGCTGGTTGGTGAAAT		[20,36]
	CH01f07a	TAMRA	CT	10	175	211	CCCTACACAGTTTCTCAACCC	CGTTTTTGGAGCGTAGGAAC		[34]
	CH05c06	ROX	GA	16		111	ATTGGAACTCTCCGTATTGTGC	ATCAACAGTAGTGGTAGCCGGT		[34]
Multiplex 2	CH04e03	FAM	(GA)n	5	179	221	TTGAAGATGTTTGGCTGTGC	TGCATGTCTGTCTCCTCCAT	60.8	[34]
	CH03g07	HEX	GA	3	195	265	AATAAGCATTCAAAGCAATCCG	TTTTTCCAAATCGAGTTTCGTT		[34]
	GD147	HEX	AG	13	121	147	TCCCGCCATTTCTCTGC	AAACCGCTGCTGCTGAAC		[33,35]

**Table 3:** Number of alleles per SSR locus across 51 pear accessions.

Locus	Na	Allele Fragment Size (bp)
CH03g07	2	206, 209
CH04e03	4	172, 204, 213, 216
GD147	3	159, 162, 167
CH01d08	3	270, 281, 303
CH01f07a	3	169, 181, 191
CH05c06	10	70, 71, 80, 87, 91, 92, 95, 100, 108, 118
EMPc11	6	128, 129, 138, 142, 149, 150
EMPc117	13	84, 85, 86, 103, 111, 113, 115, 129, 130, 131, 133, 136, 143

Na: Mean number of alleles observed per locus. Allele fragment size (bp): base pair length of each amplified allele.

**Table 4:** Allelic patterns and diversity statistics per locus across the Greek pear collection.

Mean Values		Standard Error (SE) Values	
Na	5.500	Na	1.402
Na Freq. ≥ 5%	2.625	Na Freq. ≥ 5%	0.460
Ne	2.343	Ne	0.326
I	0.953	I	0.178
No. Private Alleles	5.500	No. Private Alleles	1.402
No. LComm Alleles (≤25%)	0	No. LComm Alleles (≤25%)	0
No. LComm Alleles (≤50%)	0	No. LComm Alleles (≤50%)	0
He	0.505	He	0.075
uHe	0.510	uHe	0.076

Na: Mean number of alleles observed per locus. Na Freq. ≥ 5%: Mean number of alleles with a frequency of at least 5%. Ne: Effective number of alleles, representing genetic diversity adjusted for allele frequencies. I: Shannon’s Information Index, indicating genetic diversity. No. Private Alleles: Mean number of alleles unique to a population. No. LComm Alleles (≤25%): Mean number of alleles with a frequency less than or equal to 25%. No. LComm Alleles (≤50%): Mean number of alleles with a frequency less than or equal to 50%. He: Expected heterozygosity, representing genetic variability. uHe: Unbiased expected heterozygosity, accounting for small sample sizes.

## Data Availability

The data presented in this study are available on request from the corresponding author.
